# Renal Evaluation in Common Variable Immunodeficiency

**DOI:** 10.1155/2018/5841031

**Published:** 2018-03-15

**Authors:** Giovany Gomes Capistrano, Gdayllon Cavalcante Meneses, Fernanda Macedo de Oliveira Neves, Renata de Almeida Leitão, Alice Maria Costa Martins, Alexandre Braga Libório

**Affiliations:** ^1^Medical Sciences Postgraduate Program, Department of Clinical Medicine, Federal University of Ceará, Fortaleza, CE, Brazil; ^2^Department of Clinical and Toxicological Analysis, Faculty of Pharmacy, Federal University of Ceará, Fortaleza, CE, Brazil; ^3^Medical Course, Universidade de Fortaleza (UNIFOR), Fortaleza, CE, Brazil

## Abstract

**Introduction:**

Common variable immunodeficiency (CVID) comprises a heterogeneous group of disorders characterized by impaired antibody production. Kidney involvement in CVID is described in isolated and sporadic case reports. The objective of this study was to study the renal function pattern in CVID patients through glomerular and tubular function tests.

**Methods:**

Study of 12 patients with CVID diagnosis and 12 healthy control individuals. Glomerular filtration rate (GFR), fractional excretion of sodium (FE_Na^+^_) and potassium (FE_K^+^_), urinary concentration, and acidification capacity were measured. In addition, microalbuminuria and urinary monocyte chemoattractant protein-1 (MCP-1) were evaluated as markers of selectivity of the glomerular barrier and inflammation, respectively.

**Results:**

In relation to glomerular markers, all CVID patients had normal GFR (>90 mL/min/1.73 m^2^), and microalbuminuria and urinary MCP-1 levels were also similar to those of controls. Interestingly, CVID patients had reduced urinary concentration capacity, as demonstrated by lower *U*/*P*_Osm_ ratio, when compared to controls. Also, while all control subjects achieved a urinary pH less than 5.3, no CVID patients showed a decrease in urinary pH to such levels in response to acid loading with CaCl_2_, characterizing impaired urinary acidification capacity.

**Conclusion:**

Patients showed a trend towards an elevated prevalence of tubular dysfunction, mainly related to urinary acidification and concentration capacities.

## 1. Introduction

Common variable immunodeficiency (CVID) comprises a heterogeneous group of disorders characterized by impaired antibody provision. It is the most frequent symptomatic primary antibody disorder, with a prevalence of approximately 1 : 25,000 to 1 : 50,000 [1]. Clinically, CVID is characterized by susceptibility to recurrent infections and various sequelae and complications, including bronchiectasis, autoimmunity, enteropathy, splenomegaly, granulomatous inflammation, lymphoproliferation, and malignancy [2].

Kidney involvement in CVID has been described only sporadically in isolated case reports. Renal granuloma [3–5], focal segmental glomerulosclerosis [6], membranoproliferative glomerulonephritis [6], renal amyloidosis [7], and end-stage renal disease have all been described in CVID patients. However, it is not possible to ascertain whether all these kidney-associated clinical manifestations are directly related to CVID or only coincidental associations.

Although the most common renal diseases present with a significant reduction in glomerular filtration rate (GFR), in its acute (generally reversible) or chronic (almost always irreversible) forms, several factors can also affect renal tubular function. Renal tubular acidosis (RTA), nephrogenic diabetes insipidus, and electrolyte abnormalities are clinical entities that can go unnoticed in clinical practice, especially in their milder forms. Although overlooked by the attending physician, such disturbances can have short- and long-term complications [8, 9].

While GFR is usually calculated in daily medical practice from serum creatinine, tubular function, although altered in interstitial nephropathies such as renal granuloma, is not routinely evaluated. Because CVID has been associated with renal interstitial alterations in several cases [3–5], the objective of the present study was to study the renal function pattern in CVID patients through GFR and tubular function tests. Also, we measured urinary monocyte chemoattractant protein-1 (MCP-1) levels, an emerging biomarker to assess renal inflammatory lesions [10].

## 2. Methods

### 2.1. Patients

This is a cross-sectional study of 12 patients with CVID diagnosis, undergoing clinical follow-up at Hospital Universitário Walter Cantídio of Universidade Federal do Ceará. The study was carried out from January to July 2014. The diagnosis of CVID was based on the ESID/Pan-American Group for Immunodeficiency [11] (PAGID) criteria; patients were male or female with a marked decrease in IgG levels (≥2 SDs less than the mean for age) and a marked decrease in levels of at least 1 of the isotypes IgM or IgA, while meeting all of the following criteria:

1. onset of immunodeficiency at age older than 2 years;
2. absent isohemagglutinins, poor response to vaccines, or both;
3. exclusion of defined causes of hypogammaglobulinemia.

The patients were undergoing treatment with intravenous immunoglobulin infusion every 4 weeks. The CVID group was compared to a control group that consisted of 12 age- and sex-matched healthy volunteers.

The study protocol was reviewed and approved by the Ethics Committee of Hospital Universitário Walter Cantídio, Federal University of Ceará, in Fortaleza, Brazil. Patients were included in the study only after signing the informed consent form.

### 2.2. Clinical and Laboratory Parameters

At the medical consultation, signs and symptoms were evaluated, and the following aspects were recorded: age, gender, previous chronic diseases (heart failure, arterial hypertension, diabetes mellitus, cancer, or autoimmune diseases), recurrent urinary tract infection (≥2 episodes in six months or ≥3 in one year), time of diagnosis, use of other concomitant drugs, previous infectious complications, and their sequelae. The following laboratory parameters were evaluated: plasma and urine creatinine (*P*_Cr_ and *U*_Cr_), urea (*P*_Urea_ and *U*_Urea_), measured osmolality (*P*_Osm_ and *U*_Osm_), pH (*P*_pH_ and *U*_pH_), sodium (*P*_Na^+^_ and *U*_Na^+^_), and potassium (*P*_K^+^_ and *U*_K^+^_); plasma bicarbonate (*P*_Bic_); microalbuminuria; and urinary MCP-1.

### 2.3. Renal Function Evaluation

GFR was estimated using the CKD-EPI formula [12] and was considered abnormal when ≤90 mL/min/1.73 m^2^. All patients were submitted to a 12-hour water and food fasting. Fractional excretion of sodium (FE_Na^+^_), potassium (FE_K^+^_), and calcium (FE_Ca^++^_were calculated by standard formulas. Microalbuminuria was determined by spot collection, and abnormal values were considered if >30 mg/day. Also, urinary MCP-1, a marker of glomerular inflammation, was measured.

Urinary concentrating capacity was evaluated by the ratio between urinary and plasma osmolality (*U*/*P*_Osm_) after a 12-hour water and food fasting. Urinary acidification was evaluated by measuring urinary pH (*U*_pH_) at baseline (*T*_0_) and 4 h (*T*_4_) after ingestion of CaCl_2_, 2 mEq/kg of body weight [13]. Metabolic acidosis induced by CaCl_2_ load was documented by a decrease in serum HCO_3_– concentrations > 3 mmol/L and pH < 7.35. Failure to decrease urinary pH (*U*_pH_) to <5.3 after CaCl_2_ load was considered consistent with some form of distal renal tubular acidosis (RTA) [14]. All tubular function tests were also performed in the control group.

### 2.4. Analytical Methods

*P*_Cr_ and *U*_Cr_ were measured by the Jaffe method. *P*_Urea_ and *U*_Urea_ were measured by UV kinetic method. *P*_Na^+^_, *U*_Na^+^_, *P*_K^+^_, *U*_K^+^_, *P*_Ca^++^_, and *U*_Ca^++^_ were measured by flame photometry. Microalbuminuria was measured by the immunoturbidimetric method (Tina-quant®, Roche). *P*_Osm_ and *U*_Osm_ were assessed by freezing-point depression. *P*_pH_ and *P*_Bic_ were determined in a pH/blood gas system (AVL compact-1, Medical Instruments). *U*_pH_ was measured with a pH meter (Quims Ltda). Urinary MCP-1 was determined by sandwich enzyme-linked immunosorbent assay (ELISA) (Boster Biological Technology, Fremont, CA, USA).

### 2.5. Statistical Methods

CVID patients were compared with the control group. Fisher's exact test and chi-square test were used to analyze categorical frequencies in the patients' group. Differences between two independent continuous variables were evaluated using Student's *t*-test or Mann–Whitney test, as appropriate. Data were expressed as mean ± standard error (SE). *p* < 0.05 was considered statistically significant. SPSS software for Windows, release 10.0 (SPSS Inc. Chicago, USA) was used in all the analyses.

## 3. Results

### 3.1. Patients' Characteristics

The study included 12 unrelated adult CVID patients (7 men and 5 women; age range, 17 to 57 years; median age, 28 years), followed up at Hospital Universitário Walter Cantídio, Fortaleza, Ceará, Brazil. At the symptom onset, the median age was 16.5 years (range, 2 to 37 years). The symptoms perceived at disease onset were repeated respiratory tract infections (66.7%), persistent diarrhea (25%), and recurrent sinusitis and otitis media (8.3%). The median age at CVID diagnosis was 20 years (age range, 10 to 56 years) with a median delay of 3 years (ranging from 0 to 37 years). Four patients had splenomegaly. No patient had a diagnosis of autoimmune disease or recurrent urinary tract infection. The median time of disease duration at the time of the study was 10 years (range 1 to 38 years). All patients were undergoing regular immunoglobulin infusions. Complete demographic and clinical data of CVID patients are shown in Table 1.

### 3.2. CVID Patients Have Normal Glomerular Filtration Rate and Selective Barrier

**Figure 1:**
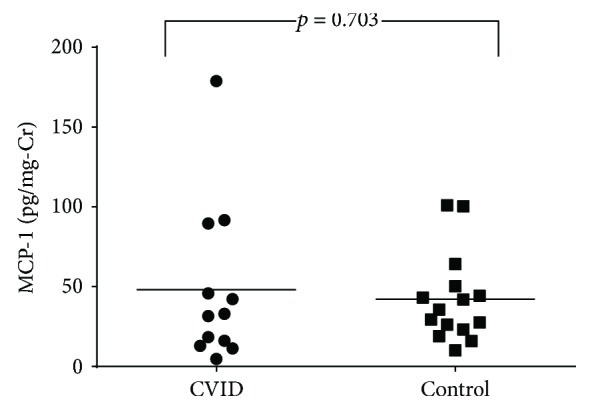
Urinary MCP-1 levels in CVID patients and control subjects.

All CVID patients had normal GFR (>90 mL/min/1.73 m^2^). The median GFR was 106 mL/min/1.73 m^2^ (range 90–212). As a measure of glomerular barrier integrity, urinary albumin excretion rate was measured, and all patients had values within the normal range. Urinary MCP-1 levels (a marker of inflammatory state—see in Discussion) were also measured, and there was a trend towards no difference when CVID patients were compared with controls (47.9 ± 14.5 versus 42.1 ± 7.1 pg/mg creatinine, *p* = 0.703); see Figure 1.

### 3.3. CVID Patients Have Selective Tubular Dysfunction

**Figure 2:**
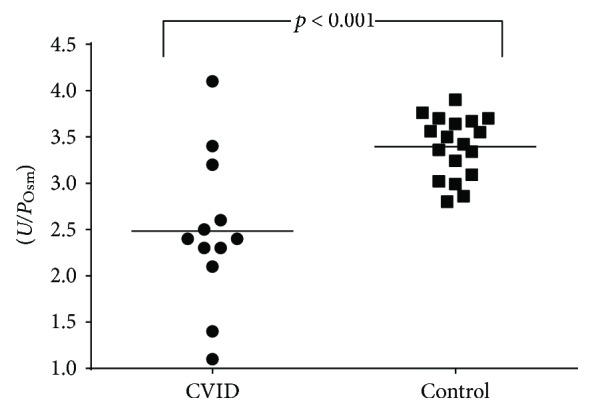
Urine/plasma osmolality (*U*/*P*_Osm_) in CVID patients and control subjects.

**Figure 3:**
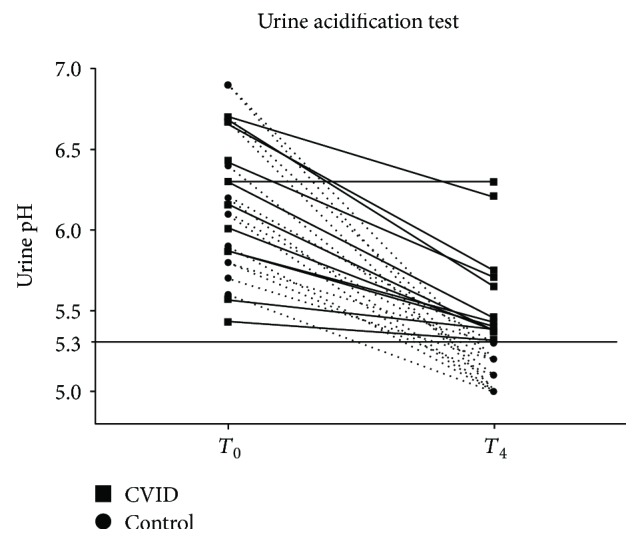
Urine pH before and after acidification test in CVID patients and controls. Urine pH under 5.3 after test is considered normal.

The comparison between the CVID patients and the control group showed no differences in age, gender, and systolic and diastolic blood pressure (Table 2). Urinary concentration capacity defect, as demonstrated by a lower *U*/*P*_Osm_ ratio, was observed when comparing CVID patients with controls—Figure 2. Regarding urinary acidification, while all control subjects achieved a urinary pH less than 5.3, no CVID patients showed a decrease in urinary pH to such levels in response to acid loading with CaCl_2_, characterizing impaired urinary acidification capacity (Figure 3). No patient had baseline serum bicarbonate levels < 22 mEq/L, and thus, a full diagnosis of tubular renal acidosis could not be attained.

Tubular handling of other electrolytes (sodium, potassium, calcium, and chlorine) was also evaluated, but all patients had serum values within normal ranges, and no significant difference in excretion fraction of such electrolytes could be detected in comparison with that of control subjects (see Table 2).

## 4. Discussion

This is the first study that evaluated glomerular and renal tubular function in CVID patients. The main finding of our study is finding that CVID showed a trend towards an association with specific functional renal tubular dysfunctions (e.g., impaired urinary concentration and acidification capacities). In fact, almost all evaluated patients presented both disturbances.

To the best of our knowledge, the only study evaluating renal disorders in CVID, in addition to case reports, is one evaluating glomerular filtration rate only, using serum creatinine-based formulas [15], and, in accordance to our findings, no significant alteration was disclosed in CVID patients regarding glomerular filtration. We expanded glomerular evaluation in such patients by measuring microalbuminuria and urinary MCP-1. While microalbuminuria is largely recognized and used in daily practice as an important risk factor in renal damage progression and therapeutic target [16], urinary MCP-1 is emerging as a potential biomarker to monitor renal inflammation and has also been associated with poor renal prognosis [10].

In our cohort, no CVID patients had glomerular filtration rate alterations or glomerular filtration barrier damage/inflammation markers. Although we studied a relatively low number of patients, we can suggest that CVID patients are at low risk of developing severe renal impairment in association with glomerular function.

However, when tubular function was assessed, we surprisingly observed a trend towards urinary acidification and concentration capacity impairment. None of the patients were able to show urinary pH reduction < 5.3 after oral acid load. To ascertain that the urinary acidification test was performed adequately, all control subjects showed adequate urinary pH reduction. Because all CVID patients had normal levels of serum bicarbonate at baseline (>24 mEq/L), we can affirm that these patients had incomplete distal renal tubular acidosis. In incomplete distal RTA, net acid excretion is maintained at a rate equal to acid generation. This is achieved by an increase in ammonium excretion that offsets the reduction in titratable acid excretion caused by the high urine pH [17]. Thus, patients with this disorder are able to maintain normal serum bicarbonate concentrations. Although CVID patients maintained normal serum bicarbonate levels, early osteoporosis [18] and recurrent renal lithiasis [19] can complicate evolution in patients with incomplete tubular renal acidosis.

The second impaired tubular function observed in CVID patients was the urinary concentration capacity. CVID patients showed lower maximum urinary concentrations after a 12 h water and food fasting when compared to control subjects. Although we did not measure serum antidiuretic hormone (ADH) levels, CVID patients' failure to achieve high urinary osmolality in comparison with controls after a 12 h water and food fasting strongly suggests there is an incomplete tubular response to ADH. Although these patients showed normal osmolality values at baseline and had no polyuria complaints, this incapacity to achieve maximum urinary concentration can predispose these patients to hypovolemia and dehydration under stress conditions.

It is a challenge to suggest any mechanisms for these tubular alterations in CVID patients, and, at present, we can only suggest possible pathways. We had previously demonstrated similar alterations in leprosy patients [20]. In leprosy, renal involvement is better described and associated with immune complex deposition. As CVID patients usually have recurrent infections, this could also be a possible explanation. Although we did not have any patients with recurrent urinary tract infection, it is possible that incipient interstitial nephropathy was present in these patients. Alternatively, it has been described that CVID patients can develop transient positivity to anti-SSA antibodies due to passive transmission via immunoglobulin infusion [21]. Because distal RTA is a common clinical feature in Sjogren's syndrome [22], it is possible that passive transmission of antibodies, such as anticarbonic anhydrase II antibody, can be related to some aspects of tubular dysfunction in such patients. Our patients did not test positive to routine autoantibody tests; however, we were not able to test them for anticarbonic anhydrase II.

The main limitation of the present study is our inability to progress regarding the pathophysiology of renal tubular disorders disclosed in CVID patients. Studies focusing on direct antibodies against renal tubular components through immunohistochemistry and additional morphological studies from renal biopsies will contribute to our understanding of renal involvement in CVID. Also, we studied a reduced number of patients, and, therefore, it is not possible to obtain definitive results.

In conclusion, we performed the most complete renal evaluation in CVID patients to date. We found no alterations in glomerular function or inflammation. However, we observed a trend towards an elevated prevalence of tubular dysfunction in CVID, mainly related to urinary acidification and concentration capacity alteration.

## Figures and Tables

**Table 1:** Common variable immunodeficiency patient's characteristics.

Patient	Age (y), sex	Time of disease (y)	Splenomegaly	Bronchiectasis	Persistent diarrhea	Urine pH *T*_4_	*U/P*_Osm_	GFR (mL/min/1.73 m^2^)	Albumin excretion rate (mg/g)	MCP-1 (pg/mg-Cr)
1	26, m	19	Yes	Yes	No	5.65	3.2	212	1.1	11.3
2	31, f	10	No	Yes	No	5.39	4.1	114	4.2	42.2
3	19, f	6	No	Yes	No	5.32	2.5	91	3.2	16.1
4	22, m	20	No	Yes	No	5.71	3.4	98	3.5	12.8
5	31, m	19	No	Yes	No	5.49	2.4	96	2.6	18.3
6	57, f	28	No	No	Yes	5.37	2.6	93	3.5	4.7
7	28, m	1	No	Yes	No	6.21	1.1	138	4.2	89.5
8	39, f	3	Yes	No	No	5.43	2.3	98	5.1	45.8
9	17, m	8	No	Yes	Yes	5.75	1.4	144	4.5	31.5
10	57, m	38	Yes	Yes	Yes	5.4	2.3	129	4.4	91.7
11	18, f	3	Yes	No	Yes	5.46	2.1	149	16	178.9
12	44, m	10	No	No	Yes	6.3	2.4	90	0.8	32.9

**Table 2:** Demographic and clinical data of CVID patients and subjects controls.

	CVID patients	Control	*p*
Age (years)	32.4 ± 4.1	31.6 ± 1.8	0.8
GFR (mL/min/1.73 m^2^)	121.0 ± 10.4	112.2 ± 2.8	0.8
Albumin/creatinine excretion rate (mg/g)	4.4 ± 1.1	3.1 ± 1.1	0.04
FE_Na^+^_ (%)	0.9 ± 0.5	0.5 ± 0.1	0.5
FE_K^+^_ (%)	7 ± 3.3	5.6 ± 0.8	0.2
FE_Ca^++^_ (%)	7.9 ± 0.4	7.4 ± 0.1	0.1
FE_Cl^−^_ (%)	1.5 ± 7.7	9.0 ± 0.1	0.4
*U*/*P*_Osm_	2.5 ± 0.2	3.4 ± 0.1	<0.001
Urinary MCP-1 (pg/mg-Cr)	48 ± 14.5	42.1 ± 7.1	0.7

FE: fractional excretion.
